# Oral manifestations of long COVID and the views of healthcare professionals

**DOI:** 10.1038/s41415-023-6715-7

**Published:** 2024-01-26

**Authors:** Dhruvi Patel, Chris Louca, Carolina Machuca Vargas

**Affiliations:** University of Portsmouth Dental Academy, William Beatty Building, Hampshire Terrace, Portsmouth, PO1 2QG, UK

## Abstract

**Introduction** Oral manifestations have been observed with this condition; however, little is known about their impact on the healthcare professionals routinely managing these patients.

**Aims** To investigate the prevalence of the oral manifestations of long COVID and the healthcare professionals' views, experiences and practices for managing these conditions.

**Materials and methods** A cross-sectional mixed-methods study, including a self-reported online questionnaire (n = 104) and healthcare professionals' (n = 7) semi-structured interviews, was conducted. Descriptive statistics and reflexive thematic analysis were used to analyse the data.

**Results** In total, 78% of respondents reported one or more oral conditions associated with COVID-19. Changes in the sense of taste and/or smell (58%), dry mouth (48.1%) and mouth sores (45.7%) were frequently reported. Themes identified from healthcare professionals' interviews were areas for research, management and interdisciplinary collaboration.

**Discussion** Allied healthcare professionals may assist dental professionals with managing oral manifestations of long COVID. Inadequate access to NHS dentistry is deterring patients worried about their oral symptoms and the limited oral knowledge of healthcare professionals affects their ability to manage patients.

**Conclusion** Healthcare professionals have observed oral manifestations in individuals diagnosed with long COVID; however, further information and training on this topic would assist them with managing patients and increasing awareness among clinicians.

**Supplementary Information:**

Zusatzmaterial online: Zu diesem Beitrag sind unter 10.1038/s41415-023-6715-7 für autorisierte Leser zusätzliche Dateien abrufbar.

## Key points

- This article provides an overview of the oral manifestations that have been observed in long COVID patients.
- Educating both patients and professionals could raise awareness of the oral manifestations of long COVID and support management in primary care.
- Inter-disciplinary collaboration would benefit patients experiencing oral manifestations of long COVID at a time when access to general dental practitioners is notably limited.

**Supplementary Information:**

Zusatzmaterial online: Zu diesem Beitrag sind unter 10.1038/s41415-023-6715-7 für autorisierte Leser zusätzliche Dateien abrufbar.

## Introduction

**Fig. 1:**
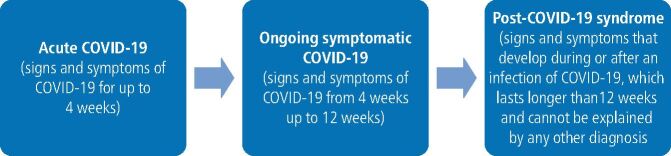
Clinical definitions of acute and long COVID^3^

The World Health Organisation declared a global pandemic caused by the SARS-CoV-2 virus, commonly known as COVID-19, in March 2020.^1^ Since then, the virus has continued to evolve, with new variants emerging to the present day. Although many people infected by COVID-19 make a full recovery, the first reports of persisting symptoms were brought to the spotlight in Spring 2020, with people self-reporting for weeks and months after contracting the initial infection.^2^ The long-term effects of COVID-19 are classified as ongoing symptomatic COVID-19 (4-12 weeks) and post-COVID-19 syndrome (over 12 weeks), commonly known as ‘long COVID' (Fig. 1).^3^

Breathlessness, fatigue, heart palpitations and brain fog are a few of the growing list of long COVID symptoms.^2^ The Office for National Statistics reported that as of October 2022, approximately 3.3% of the population (2.1 million people) in the UK are experiencing long COVID, including children.^4^^,^^5^

Oral manifestations of COVID-19 have been reported sporadically in the literature (Box 1) and include ulcers, xerostomia, depapillated tongue and leukoplakia, to name a few.^6^^,^^7^

Although oral manifestations have been reported in patients diagnosed with long COVID, information and guidance available for healthcare professionals managing these patients is limited. Long COVID services have been established in the UK following the publication of the National Institute of Health and Care Excellence guidance on managing the long-term effects of COVID-19.^3^ These services vary across the country and involve a multidisciplinary approach to managing patients. However, the guidance does not include management of patients with oral symptoms.^3^

Therefore, the prevalence of oral manifestations of long COVID and the experiences of healthcare professionals in managing these conditions were investigated in order to acquire information that can be used to improve existing services and enhance the patient experience.

Box 1 Oral manifestations of acute and long COVID^6^^,^^7^

- Aphthous ulcers
- Blisters
- Necrotising gingivitis
- Herpetic ulcers
- Depapillated tongue
- Angular cheilitis
- Erythema multiforme-like lesions
- White plaques
- Dark pigmentations
- Facial pain
- Oral submucous fibrosis
- Burning mouth
- Xerostomia
- Dysgeusia
- Candidiasis
- Vasculitis
- Mucositis
- Kawasaki like lesions
- Melkersson-Rosenthal syndrome
- Necrotising periodontitis
- Angina bullosa like lesions
- Atypical sweet syndrome
- Enanthem
- Leukoplakia
- Facial nerve palsy
- Ageusia
- Erosion
- Bullae
- Vesicles
- Pustule
- Fissured tongue
- Macule
- Papule
- Halitosis
- Haemorrhagic crust
- Petechia
- Necrosis
- Swelling
- Erythema

## Methodology

Change this paragraph to read: 

This was a cross-sectional mixed methods study with two elements: a self-reported online questionnaire and semi-structured interviews of healthcare professionals (see Supplementary Information for details of the questionnaire and interview guide).

From June to July 2021, an online questionnaire was shared among three, private, UK-based Facebook COVID-19 support groups: COVID-19 Research Involvement Group; Portsmouth Coronavirus Support Group; and Gosport and Fareham COVID-19 Support and Information Group. The online survey collected demographic information, acute and long-lasting symptoms of COVID-19, oral health conditions manifested while infected with COVID-19 and impacts of oral health on daily life.

The qualitative component involved recruiting a diverse sample of healthcare professionals from different speciality areas and varying scope of practice to provide an overall view of the experience of healthcare professionals with patients experiencing oral conditions due to long COVID. Participants were initially recruited via the research team's professional networks and then by a snowball process, whereby new participants were recruited by previous participants sharing details of the study. Email invitations were sent to numerous GP practices (general medical practitioners), medical specialities within hospital departments and long COVID clinics in Wessex.

Following the recommendations for reflexive thematic analysis, theoretical saturation was used to determine the sample size.^8^ Data analysis was conducted simultaneously with data collection and the recruitment stopped when there was adequate information to map out the local healthcare service provision and gain an overall view of the health professionals' experiences.

The interviews were conducted between February and May 2022 via video conferencing software or in person, audio-recorded and transcribed verbatim. Data relevant to the research topic were identified initially before being selectively organised based on relevant key themes.^9^ The overarching themes were recorded with respect to the research objectives. Themes were reviewed independently by two members of the research team for validation. Data were anonymised and SPSS and NVivo 12 Plus software were used for analysis.

Ethical approval was obtained from the University of Portsmouth Science and Health Faculty Ethics Committee (SHFEC 2022-007). All participants consented to participate in the study and to have their data used as part of the research. Written consent was obtained for the publication of verbatim quotes from the participants that were interviewed.

## Results

A total of 104 adult participants responded to the online questionnaire, of which 89.4% were white British women. Most of the participants were 35-49 years of age (41.3%). In total, 55% rated their health as fair or poor, with 73% reporting one or more medical condition (Table 1).

**Table 1:** Demographic characteristics of the sample

Characteristics	N (104)	%
**Sex**		
Female	93	89.4
Male	8	7.7
Other	3	2.9
**Age**		
18-24	4	3.8
25-34	18	17.3
35-49	43	41.3
50-64	35	33.7
65-79	4	3.8
**Ethnicity**		
White - British, Irish, other	93	89.4
Asian/Asian British - Indian, Pakistani, Bangladeshi, other	5	4.8
Multiracial - white and Black/Black British	1	1
Multiracial - other	3	2.9
Middle Eastern/Middle Eastern British - Arab, Turkish, other	1	1
Other ethnic group	1	1
**Medical conditions**		
None	28	26.9
One or more	76	73
**General health rating**		
Poor	28	26.9
Fair	29	27.9
Good	26	25
Very good	15	14.4
Excellent	6	5.8
**Oral health conditions during COVID-19**		
None	32	30.7
One or more	72	69.2
**Oral symptoms duration during COVID-19**		
More than 3 months	49	47.1
1-3 months	4	3.8
2 weeks to a month	8	7.7
1-7 days	11	10.6
N/A	32	30.7
**Oral health rating**		
Very poor	2	1.9
Poor	41	39.4
Good	39	37.5
Very good	16	15.4
Excellent	6	5.8

**Fig. 2:**
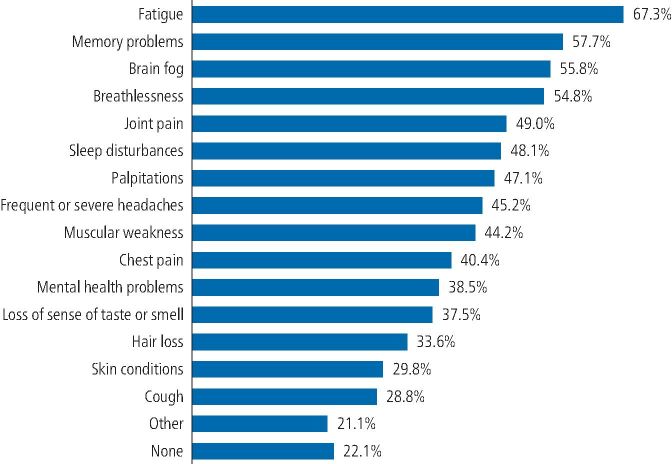
COVID-19 symptoms reported four weeks after the initial infection

Overall, 78 (75%) participants reported complications lasting more than four weeks after the initial COVID-19 infection. Fatigue (86.4%), memory problems (74%), brain fog (71.6%) and shortness of breath (70.4%) were the most frequent symptoms reported by participants (Fig. 2).

**Fig. 3:**
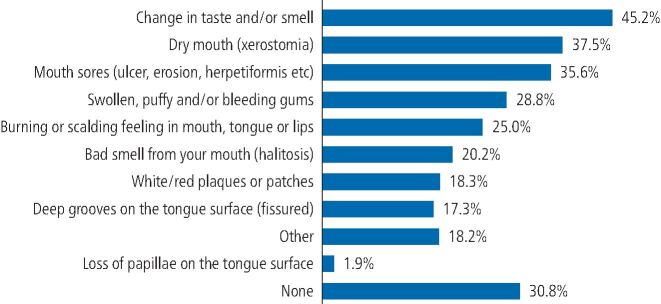
Oral symptoms reported in COVID-19 participants

Moreover, 69% of respondents reported one or more oral conditions associated with COVID-19, with 47.1% reporting symptoms lasting more than three months (Table 1). Most of the participants had changes in the sense of taste and/or smell (58%), dry mouth (48.1%) and mouth sores (45.7%) (Fig. 3).

Only 27.2% had seen a dentist for their oral symptoms, despite 40.4% rating their oral health as poor or very poor. Moreover, participants reported unrestored teeth (14.4%) and teeth with mobility or gum problems (18.3%), and 35% reported painful aching in their mouth as fairly and very often (Table 2).

**Table 2:** Oral health impacts on daily life

**How often during the last year...**	**Very often (%)**	**Fairly often (%)**	**Occasionally (%)**	**Hardly ever (%)**	**Never (%)**
Have you had painful aching anywhere in your mouth?	16.3	18.3	26	14.4	25
Have you felt that life in general was less satisfying because of problems with your teeth, mouth or dentures?	8.7	12.5	23.1	19.2	36.5
Have you had difficulty doing your usual jobs because of problems with your teeth, mouth or dentures?	1.9	3.8	18.3	17.3	58.7
Have you felt that your sense of taste has worsened because of problems with your teeth, mouth or dentures?	5.8	4.8	19.2	16.3	53.8
Have you avoided particular foods because of problems with your teeth, mouth or dentures?	13.5	11.5	18.3	21.2	35.6
Have you been a bit embarrassed because of problems with your teeth, mouth or dentures?	11.5	11.5	17.3	14.4	45.2
Have you found it uncomfortable to eat any foods because of problems with your teeth, mouth or dentures?	17.3	11.5	16.3	17.3	37.5

Seven participants from a range of disciplines were interviewed: three speech and language therapists, one respiratory physiotherapist, one dental hygienist, one oral and maxillofacial surgeon and one staff grade in oral surgery.

The overarching themes established following data analysis were areas for research, management and inter-disciplinary collaboration.

### Areas for research

Due to the lack of information available on the oral manifestations of long COVID, all participants identified areas for further research that they felt could contribute to their current knowledge and could guide them on how to manage these patients in the future. The prevalence of oral conditions seen in long COVID patients was mentioned by everyone, with anecdotal values of 5-10% being reported by some participants. Participants felt it was important to establish whether there is a causational relationship between long COVID and the presence of oral manifestations:

- ‘*So I don't know if it's caused by long COVID, or it's just become more common because they've changed their habits you know? Maybe they're too tired at the end of the day to kind of really get in, keep their mouth clean, or are they more sensitive?*' (participant 6).

Allied healthcare professionals were not always able to name the conditions using medical terminology or offer the patient a clinical diagnosis, as the oral cavity is not their area of clinical expertise. However, patients are signposted to seek advice from a dentist or their general practitioner. The oral conditions most observed by participants were ‘oral thrush', xerostomia, ‘COVID tongue', recurrent oral ulcers, gingival infections and temporomandibular joint dysfunction issues:

- ‘*And I'd say maybe 1 in 15, 1 in 20, are then talking about, you know, their oral health is much worse. They've got furrows on their tongue, infections in their gums. They're talking about worse teeth*' (participant 6).

There was considerable discussion about clinical diagnosis. Allied healthcare professionals in particular were unsure of who should make the diagnosis. There was also a general feeling that currently, the diagnosis is made by exclusion based on the symptom history reported by the patient and the appearance of the oral manifestations in relation to when the initial COVID infection occurred. It was pointed out that no special investigation exists to confirm that the symptoms are directly caused by the COVID-19 infection:

- ‘*But usually, if it's long COVID, it will either be as a direct result of their initial infection, so it will be immediate, or we're finding there's a bit of a gap between the initial infection and them coming down with long COVID, which can be kind of about six weeks or so'* (participant 1).

### Management

The importance of both patient and professional education was emphasised by the participants, with suggestions of plausible teaching methods. These ranged from the production of patient information leaflets to organising webinars and training sessions for professionals. Participants felt that specialists in the field should be leading in educating others and clinicians should be informed of where to seek further advice from:

- ‘*It's teaching the patient how to manage their symptoms. And I think that managing your mouth symptoms could be a part and parcel of that, okay, which is why I think in general the dentist would be a really good point of contact'* (participant 7).

There was some debate and deliberation regarding which speciality would be most suitable to manage oral conditions in long COVID patients, and all participants nominated dentists, with additional specialist support where required:

- *‘I think it ideally would be the general dental practitioner. Really, I think once all else has been excluded, I think the patient should feel comfortable going to their general dental practitioner who'd be able to give them advice on, you know, methods to keep things comfortable and how to'* (participant 7).

### Inter-disciplinary collaboration

The number of different healthcare professionals involved in the care of a single patient was highlighted by participants, as they listed the different specialities and services that patients may encounter to manage long COVID. This included, but was not limited to, general medical practitioners, cardiologists, rheumatologists and physiotherapists.

As a result of the myriad of health issues experienced by long COVID patients, it was pointed out that patients may already be under the care of multiple specialities and have numerous pending appointments, so arranging an additional appointment for the patient's oral conditions may be burdensome:

- ‘*… with disability does sometimes come economic effects of disability, so a lot of people now suddenly are in the position where they couldn't possibly pay for private dentistry because they're not working due to fatigue and everything*' (participant 6).

Patients may be managed in primary or secondary care, community clinics, or GP practices and some participants pondered whether a healthcare professional already involved in their care could assist with managing oral symptoms:

- ‘*…within speech therapy, we can look at generally how to promote mouth care and make sure that your mouth is clean. But some of these symptoms that are cropping up, I'm not too sure really*' (participant 2).

Participants discussed the difficulties they have experienced when referring patients to other specialities for second opinions or management due to the increasing waiting times across the NHS and the helplessness they feel as a consequence:

- ‘*So if there are simple, straightforward things then that can stay with us because we can be educated on advice for and [sic] things like that, that's fine. They can stay within the service so that we're not just adding to other people's waiting lists'* (participant 1).

A compounding factor is the well-documented lack of access to NHS dentists, which has recently been reported by the media and was alluded to by all participants, plus the absence of a direct referral pathway to a local NHS dentist.^10^ Consequently, healthcare professionals can only signpost and advise patients with oral and dental issues to register with a dentist:

- ‘*…we're seeing less and less access to dentistry. Because we used to be able to get, you know, refer people to dentists, and now we just can't. Even for oral hygiene*' (participant 6).

## Discussion

The demographic of the respondents of the online survey aligns closely with those with the highest prevalence of self-reported long COVID in the UK, which is women, 35-69 years of age, with an existing health condition or disability that impacts their daily life.^4^ The most frequently lasting symptoms reported by the respondents also mirror national statistics, with fatigue being experienced by 60% of people living with the condition.^4^

The data suggest that the oral manifestations reported by the survey respondents and those observed by healthcare professionals are in keeping with those previously reported in the literature; however, the assessment and management of patients varied depending on the clinical role of the participant.^6^^,^^7^ Referral of patients was inconsistent, as no pathway or guidelines currently exist, which leads to variable outcomes for patients.

The inconsistencies in management were demonstrated on an individual basis, as patients were referred to different specialities by different clinicians; although, the dentist was the practitioner of choice for managing these patients. Although the online survey sample size was limited, a significant number of respondents reported one or more oral conditions associated with long COVID, which advocates the benefits of raising awareness of these among healthcare professionals. The important role of dentists in managing oral health to minimise the impact on the individual has been emphasised for other diseases, such as cardiac conditions, yet dentistry is still not integrated consistently as a speciality forming the multidisciplinary team.^11^^,^^12^

The survey respondents' admittance that they would deem their oral health to be poor or very poor highlights the regrettable reality for a considerable proportion of the UK population who are struggling to access oral care.^10^ Healthcare professionals are further deterred from referring patients to a dentist due to the current state of NHS dentistry in the UK. Current figures show that accessing an NHS dentist is near enough impossible in many parts of the country, and with a looming economic crisis, private treatment may not be an affordable option for patients.^10^ NHS Digital reported that in the quarter ending June 2022, only 36.9% of adults in England had received NHS dental care in the preceding 24 months, compared with 49.6% pre-pandemic in March 2020.^13^ This disenfranchises long COVID patients, who require support with managing their oral symptoms but are struggling to access care in primary dental care settings.

Allied healthcare professionals can provide patients with basic mouth care advice and reassurance. However, they highlighted a lack of clarity regarding the best place to access evidence-based resources that they could signpost patients to, and suggested that a tailored patient information leaflet and further resources addressing the other common symptoms would be beneficial.

Furthermore, there was confusion regarding the scope of practice of various oral and dental specialities, as participants pondered whether these patients should be seen by a specific speciality, and if so, which one. As no referral pathway or guidance is available, this could further delay the patient receiving care or lead to the patient being passed between specialities without resolution. In addition, there are numerous specialities which could be involved, such as oral medicine, oral surgery and oral and maxillofacial surgery; however, co-ordinating them to accommodate this group of patients during a time when the NHS workforce is endeavouring to manage the excessively high waiting lists poses a challenge in itself.^14^

Some oral manifestations reported by participants of this study include changes to the tongue, both clinical and sensory, supporting the findings of the ZOE COVID study; although, the oral manifestations are rare when compared with other systemic symptoms.^15^ Nonetheless, there is limited information available regarding geographic tongue and the possible causes of tongue symptoms.

Treatment modifications that dentists should consider due to drug interactions with medication(s) that patients are prescribed to manage their systemic long COVID symptoms have been identified, along with the impact of fatigue and brain fog on their daily functioning.^16^ Other research investigating the prevalence of oral manifestations of COVID-19 overwhelmingly focuses on the conditions observed in the acute phase and merely mention that for some, symptoms are slower to resolve.^17^ Healthcare professionals would benefit from clarity regarding which oral manifestations most commonly persist in long COVID, in parallel with the oral manifestations occurring in other systemic diseases, as this would encourage them to consider long COVID as a clinical diagnosis after frequent causes have been excluded.^18^

The generalisability of the results is limited, as the participants for both elements of the study were based in the Wessex region. With its demographic nuances, the findings cannot reliably represent the national picture. As participants volunteered to partake in the research and had personal experiences of either oral conditions associated with COVID infection, or of managing patients with oral manifestations of long COVID, their individual biases must be considered when analysing the data, and again, may not reflect the views of their peers and colleagues. Although data saturation was reached very quickly, the findings may not be replicated in other parts of the country, as all long COVID services operate independently, so it could be the case that some centres have dental professionals present to assess and manage these patients. The findings do not account for general dental practitioners already confidently managing long COVID patients with oral manifestations; however, there are no data available at present showing proportionately where these patients are being cared for.

The findings are yet another example of oral health not being integrated into the management of systemic conditions, despite the rapidly increasing recognition of the impact of poor oral health on the quality of life.^19^ Further research is required to ascertain the prevalence of oral manifestations of long COVID and the specific conditions commonly seen, along with the severity and complexity of the conditions, which would be a key indicator in agreeing on which healthcare professionals can manage this condition and setting up referral pathways where further support is required. Given the limited access to NHS dentists and patients being subjected to long waiting times for an initial consultation, there is an argument for a referral pathway to be instated, or for a dental professional representative in the long COVID multidisciplinary team.

## Conclusion

Oral manifestations of long COVID will continue to exist for as long as the disease does; however, the prevalence remains unknown. Healthcare professionals should be made aware of its existence so that they can develop their clinical practice. Currently, clinicians are managing these patients to the best of their ability, albeit disjointedly, as there is a lack of clinical guidance outlining the specific oral conditions frequently observed, how and who should manage them, and which cases, if any, require referral to a specialist. While patients endure increasing difficulty in accessing routine dental care, allied healthcare professionals may play an important role in managing this cohort of patients, although they would require further training to do so.

### Supplementary Information

Supplementary Information (PDF 383KB)
