# Supplementary material for: Oral manifestations of long COVID and the views of healthcare professionals
Source: Br Dent J. 2024 Jan 26;236(2):111–6. doi: 10.1038/s41415-023-6715-7 (PMC10817823; doi:10.1038/s41415-023-6715-7)
Supplement: Supplementary file 1 — Supplementary Information (PDF 383KB) [file 41415_2023_6715_MOESM1_ESM.pdf]

Semi-structured Interview Guide

Title: Oral manifestations of long COVID and the experience of healthcare professionals.

Preamble

Interviewer introduction (name, role, study principal investigator) and the study (review objective).

Initial Engagement

1. Have you come across patients with oral conditions due to long COVID in your clinical practice so far?
    - If so, how many patients of this description have you managed?

2. How did the patient present to you? i.e. was the patient referred to you from another practitioner or were you the primary contact?

3. What were the oral manifestations these patients presented with?
    - Probe to provide specific examples or descriptions
    - Diagnoses or differential diagnoses given

4. Was(were) the oral symptom(s) described by the patient their primary complaint of long COVID?
    - Did the patient report other long COVID symptoms they were experiencing along with the oral symptoms?
    - More information on the other symptoms – the nature of them and if there was any mention of who's care the patient was under to manage these

Topic 1: Assessment and management of patients presenting with oral manifestations of long COVID

5. How did you determine that the oral symptoms were directly caused by long COVID, and exclude other possible diagnoses?

6. How have you managed patients presenting with oral manifestations of long COVID so far?
    - Special investigations
    - Medications prescribed
    - Frequency of follow-up appointments
    - Discharge patient? Transfer care to primary care practitioner?
    - Involvement of other specialty areas

- Did the patients' symptoms fully recover?

7. Do you feel confident in managing these patients?
    - If yes then why?
    - If no then why not?

8. Were there any cases where you were unable to provide the patient with a diagnosis for their symptoms?
    - Probe to find out whether the case was discussed with colleagues or referred for a second opinion. Where was the patient referred to?

9. As the COVID pandemic continues, do you think that healthcare professionals should be made aware of oral manifestations of long COVID?
    - Why do you think it is important for other professionals to know?
    - Give examples of things they would have liked to have known before encountering the condition
    - What do you think would be the best way to increase awareness?
    - What information would you like to find out regarding patients with oral manifestations of long COVID?
    - Any changes to clinical practice

Topic 2: Patient referral pathways

10. Who do you feel is the most appropriate clinician to manage these patients?
    - Why?

11. Are you aware of care pathways for long COVID patients?
    - If yes, how was this brought to your attention?

12. What services do you feel long COVID patients with oral symptoms would benefit from?
    - Probe to give examples and explain why
    - If they feel existing services are sufficient, then again explain why

13. As a healthcare professional, what support do you require to manage these patients?

14. Do you have any final thoughts?

Thank you for participating in our COVID-19 study. Your perspectives and experiences are appreciated.

Oral Manifestations of COVID-19 study (ORION C-19)

* Indicates required question

The ORION C-19 study investigate the characteristics of oral manifestations of COVID-19 at different stages of the disease. We are looking for volunteers who are willing to provide anonymous information about their oral health and how COVID-19 has affected their quality of life. People taking part in the study will be asked to respond an electronic questionnaire to collect information. The questionnaire takes about 10 minutes to complete. Participation in this study is voluntary and you are free to withdraw at any time without giving any reason before submitting your responses. NO personal or identifiable information will be collected. All information will be kept strictly confidential. Data will be processed in accordance with the General Data Protection Regulation 2016 (GDPR).

1. I confirm that I have read and understand the information for the ORION C-19 study. I have had the opportunity to consider the information, ask questions and have had these answered satisfactorily. *

   Mark only one oval.

   - Yes
   - No

We would like to start by asking some general questions about you

2. What is your gender?

   Mark only one oval.

   - Male
   - Female
   - Prefer not to say
   - Other: ____________________

3. What is your age?

Mark only one oval.

- ( ) 18-24
- ( ) 25-34
- ( ) 35-49
- ( ) 50-64
- ( ) 65-79
- ( ) 80+

4. What is your ethnicity?

Mark only one oval.

- ( ) Asian/Asian British- Indian, Pakistani, Bangladeshi, other
- ( ) Black/Black British-Caribbean, African, other
- ( ) Mixed race- White and Black/Black British
- ( ) Mixed race- other
- ( ) White- British, Irish, other
- ( ) Chinese/Chinese British
- ( ) Middle Eastern/ Middle Easter British- Arab, Turkish, other
- ( ) Other ethnic group
- ( ) Prefer not to say

5. What is your relationship status?

Mark only one oval.

- ( ) Single, never married
- ( ) Single, divorced or widowed
- ( ) In a relationship/ married but living apart
- ( ) In a relationship/ married and cohabiting

6. What is your highest educational attainment?

Mark only one oval.

- ◯ No qualifications
- ◯ Completed GCSE/CSE/O-levels or equivalent (at school till aged 16)
- ◯ Completed post-16 vocational course
- ◯ A-levels or equivalent (at school till aged 18)
- ◯ Undergraduate degree or professional qualification
- ◯ Postgraduate degree

7. What type of place do you live?

Mark only one oval.

- ◯ House
- ◯ Room(s) in a shared house (e.g. as a lodger)
- ◯ Flat in a flat block
- ◯ Student halls
- ◯ Residential home
- ◯ Other: ______________________

8. Which describes the home you live in?

Mark only one oval.

- ◯ Owned outright
- ◯ Owned with the help of a mortgage
- ◯ Shared ownership (part owned, part rented)
- ◯ Rented
- ◯ Living rent free

9. What is your employment status?

Mark only one oval.

- Still at school
- At university
- Self employed
- In part-time employment
- In full-time employment
- Unable to work due to disability
- Homemaker/ Full time parent
- Unemployed and seeking work
- Retired

We would like to know more about your health

10. In general, would you say your health is:

Mark only one oval.

- Excellent
- Very good
- Good
- Fair
- Poor

11. Do you have any of the following medical conditions?

Mark only one oval.

- High blood pressure
- Diabetes
- Heart disease
- Lung disease (e.g. asthma or COPD)
- Cancer
- Another clinically-diagnosed chronic physical health condition
- Clinically-diagnosed depression
- Clinically-diagnosed anxiety
- Another clinically-diagnosed mental health problem
- No. I don't have any medical condition
- Not sure

12. Do you smoke?

Mark only one oval.

- Non-smoker
- Ex-smoker
- Current light smoker (9 or less a day)
- Current moderate smoker (10-19 a day)
- Current heavy smoker (20+ a day)

13. How many alcoholic drinks have you had in the past week (e.g. how many glasses of wine/ pints of beer or cider/ shots of spirits)?

Mark only one oval.

- 1
- 2
- 3
- 4
- 5 or more

14. How many days last week did you do moderate or vigorous physical activity (activity that raises your heart rate and makes your breathe faster and feel warmer) for 15 minutes or more?

Mark only one oval.

- 1
- 2
- 3
- 4
- 5
- 6
- 7

We would like to know more about your COVID-19 experience

15. Have you been swab tested for COVID-19, commonly referred to as coronavirus?

Mark only one oval.

- Yes, and I was positive
- Yes, and I was negative
- Yes, and my results are pending
- No, but I have had flu-like symptoms with a fever at some point
- No, and I have not had flu-like symptoms with a fever at some point

COVID-19 Symptoms

16. Have you had any of the following symptoms at some point of the disease? Select all that apply

Tick all that apply.

- [ ] None
- [ ] Fever
- [ ] Shortness of breath
- [ ] Dry cough
- [ ] Nasal congestion
- [ ] Runny nose
- [ ] Sore throat
- [ ] Feeling tired or fatigue
- [ ] Chills
- [ ] Body aches
- [ ] Headache
- [ ] Cough producing phlegm
- [ ] Abdominal pain
- [ ] Nausea or vomiting
- [ ] Diarrhoea
- [ ] Other: ______________________

17. How long did these symptoms last?

Mark only one oval.

- ( ) Less that 1 day
- ( ) 1-7 days
- ( ) 2 weeks to a month
- ( ) 1-3 months
- ( ) More than 3 months

18. Have you had any of the following lesions in your lips, mouth, tongue, gingiva, oropharynx or tonsils at some point of the disease? Select all that apply

Tick all that apply.

- [ ] None
- [ ] Ulcer, erosion, herpetiform or aphthous-like lesions
- [ ] White/red plaques or patches
- [ ] Swollen, puffy and/or bleeding gums
- [ ] Fissured or depapillated tongue
- [ ] Burning or scalded feeling in mouth, tongue or lips
- [ ] Bad smell from your mouth (e.g. halitosis)
- [ ] Change in taste and/or smell

19. How long did these symptoms last?

Mark only one oval.

- ( ) Less that 1 day
- ( ) 1-7 days
- ( ) 2 weeks to a month
- ( ) 1-3 months
- ( ) More than 3 months

COVID-19 hospitalisation

20. Were you hospitalised due to these COVID-19 symptoms?

Mark only one oval.

- ( ) Yes
- ( ) No
- ( ) Not sure

21. How long were you hospitalised?

Mark only one oval.

- Less that 1 day
- 1-7 days
- 2 weeks to a month
- 1-3 months
- More than 3 months

22. Were you hospitalised in the Intensive Care Unit (ICU) with a ventilator?

Mark only one oval.

- Yes
- No
- Not sure

23. Were you hospitalised in the Intensive Care Unit (ICU) with oxygen?

Mark only one oval.

- Yes
- No
- Not sure

24. Have you had any of the following complications due to your COVID-19 illness? Select all that apply

Tick all that apply.

- [ ] None
- [ ] Pneumonia
- [ ] Severe pneumonia leading to hospitalisation
- [ ] Respiratory failure
- [ ] Septic shock
- [ ] Multiple organ dysfunction or failure
- [ ] Blood clots
- [ ] Stroke
- [ ] Other: ______________________

How much are you currently affected in your everyday life by COVID-19?

25. Please indicate which one of the following statements applies to you most:

Mark only one oval.

- ◯ I have no limitations in my everyday life and no symptoms, pain, depression or anxiety
- ◯ I have negligible limitations in my everyday life as I can perform all usual duties/activities, although I still have persistent symptoms, pain, depression or anxiety
- ◯ I suffer from limitations in my everyday life as I occasionally need to avoid or reduce usual duties/activities or need to spread these over time due to symptoms, pain, depression or anxiety. I am, however, able to perform all activities without any assistance
- ◯ I suffer from limitations in my everyday life as I am not able to perform all usual duties/activities due to symptoms, pain, depression or anxiety. I am, however, able to take care of myself without any assistance
- ◯ I suffer from severe limitations in my everyday life: I am not able to take care of myself and therefore I am dependent on nursing care and/or assistance from another person due to symptoms, pain, depression or anxiety

26. During the past 4 weeks, how much did physical health problems limit your usual physical activities (such as walking or climbing stairs)?

Mark only one oval.

- Not at all
- Very little
- Somewhat
- Quite a lot
- Could not do physical activities

27. During the past 4 weeks, how much difficulty did you have doing your daily work, both at home and away from home, because of your physical health?

Mark only one oval.

- Not at all
- A little bit
- Some
- Quite a lot
- Could not do daily work

28. How much bodily pain have you had during the past 4 weeks?

Mark only one oval.

- None
- Very mild
- Mild
- Moderate
- Severe
- Very severe

29. During the past 4 weeks, how much energy did you have?

Mark only one oval.

- Very much
- Quite a lot
- Some
- A little
- None

30. During the past 4 weeks, how much did your physical health or emotional problems limit your usual social activities with family or friends?

Mark only one oval.

- Not at all
- Very little
- Somewhat
- Quite a lot
- Could do social activities

31. During the past 4 weeks, how much have you been bothered by emotional problems (such as feeling anxious, depressed or irritable)?

Mark only one oval.

- Not at all
- Slightly
- Moderately
- Quite a lot
- Extremely

32. During the past 4 weeks, how much did personal or emotional problems keep you from doing your usual work, school or other daily activities?

Mark only one oval.

- Not at all
- Very little
- Somewhat
- Quite a lot
- Could not do daily activities

How much are you currently affected in your everyday life by the oral manifestations of COVID-19?

33. Overall, how would you rate the health of your mouth, teeth and gums

Mark only one oval.

- Excellent
- Very good
- Good
- Poor
- Very poor

34. Are there restorations in your teeth?

Mark only one oval.

- No restorations at all
- 1-2 restorations
- 3 restorations or more
- Don’t know

35. Do you have any unrestored decayed teeth?

Mark only one oval.

- Yes
- No
- Don't know

36. Do you have teeth with mobility?

Mark only one oval.

- Yes
- No
- Don't know

37. Have you had trouble pronouncing any words because of problems with your teeth, mouth or dentures?

Mark only one oval.

- Very often
- Fairly Often
- Ocassionally
- Hardly ever
- Never

38. Have you felt that your sense of taste has worsened because of problems with your teeth, mouth or dentures?

Mark only one oval.

- Very often
- Fairly Often
- Ocassionally
- Hardly ever
- Never

39. Have you had painful aching in your mouth?

Mark only one oval.

- Very often
- Fairly Often
- Ocassionally
- Hardly ever
- Never

40. Have you found it uncomfortable to eat any foods because of problems with your teeth, mouth or dentures?

Mark only one oval.

- Very often
- Fairly Often
- Ocassionally
- Hardly ever
- Never

41. Have you felt self conscious because of problems with your teeth, mouth or dentures?

Mark only one oval.

- Very often
- Fairly Often
- Ocassionally
- Hardly ever
- Never

42. Have you felt tense because of problems with your teeth, mouth or dentures?

Mark only one oval.

- Very often
- Fairly Often
- Ocassionally
- Hardly ever
- Never

43. Has your diet been unsatisfactory because of problems with your teeth, mouth or dentures?

Mark only one oval.

- Very often
- Fairly Often
- Ocassionally
- Hardly ever
- Never

44. Have you had to interrupt meals because of problems with your teeth, mouth or dentures?

Mark only one oval.

- Very often
- Fairly Often
- Ocassionally
- Hardly ever
- Never

45. Have you found it difficult to relax because of problems with your teeth, mouth or dentures?

Mark only one oval.

- Very often
- Fairly Often
- Ocassionally
- Hardly ever
- Never

46. Have you been a bit embarrassed because of problems with your teeth, mouth or dentures?

Mark only one oval.

- Very often
- Fairly Often
- Ocassionally
- Hardly ever
- Never

47. Have you been a bit irritable with other people because of problems with your teeth, mouth or dentures?

Mark only one oval.

- Very often
- Fairly Often
- Ocassionally
- Hardly ever
- Never

48. Have you had difficulty doing your usual jobs because of problems with your teeth, mouth or dentures?

Mark only one oval.

- Very often
- Fairly Often
- Ocassionally
- Hardly ever
- Never

49. Have you felt that life in general was less satisfying because of problems with your teeth, mouth or dentures?

Mark only one oval.

- Very often
- Fairly Often
- Ocassionally
- Hardly ever
- Never

50. Have you been totally unable to function because of problems with your teeth, mouth or dentures?

Mark only one oval.

- Very often
- Fairly Often
- Ocassionally
- Hardly ever
- Never

This content is neither created nor endorsed by Google.

Google Forms
